# Mating avoidance in female olive baboons (*Papio anubis*) infected by *Treponema pallidum*

**DOI:** 10.1126/sciadv.aaw9724

**Published:** 2019-12-04

**Authors:** F. M. D. Paciência, J. Rushmore, I. S. Chuma, I. F. Lipende, D. Caillaud, S. Knauf, D. Zinner

**Affiliations:** 1 Cognitive Ethology Laboratory, Leibniz Institute for Primate Research, German Primate Center, Kellnerweg 4, 37077 Göttingen, Germany.; 2 Work Group Neglected Tropical Diseases, Infection Biology Unit, Leibniz Institute for Primate Research, German Primate Center, Kellnerweg 4, 37077 Göttingen, Germany.; 3 EpiCenter for Disease Dynamics, One Health Institute, School of Veterinary Medicine, University of California, Davis, One Shields Avenue, Davis, CA 95616, USA.; 4 Sokoine University of Agriculture, P.O. Box 3000, Chuo Kikuu, Morogoro, Tanzania.; 5 Department of Anthropology, University of California, One Shields Avenue, Davis, CA 95616, USA.; 6 Division of Microbiology and Animal Hygiene, Georg-August-University, Göttingen, Germany.; 7 Leibniz ScienceCampus Primate Cognition, Göttingen, Germany.

## Abstract

Sexually transmitted infections (STIs) are ubiquitous within wild animal populations, yet it remains largely unknown whether animals evolved behavioral avoidance mechanisms in response to STI acquisition. We investigated the mating behavior of a wild population of olive baboons (*Papio anubis*) infected by the bacterium *Treponema pallidum*. This pathogen causes highly conspicuous genital ulcerations in males and females, which signal infectious individuals. We analyzed data on 876 mating attempts and associated acceptance or rejection responses in a group of about 170 baboons. Our findings indicate that females are more likely to avoid copulation if either the mating partner or females themselves have ulcerated genitals. We suggest that this outcome is linked to the overall higher choosiness and infection-risk susceptibility typically exhibited by females. Our results show that selection pressures imposed by pathogens induce individual behavioral modifications, leading to altered mate choice and could reduce promiscuity in a wild nonhuman primate population.

## Teaser

Genital ulcers caused by *Treponema* *pallidum* lead to mating avoidance in female olive baboons..

## INTRODUCTION

Infectious diseases are pervasive in the animal kingdom and pose a serious threat to many wildlife populations (*1*). Fitness costs associated with infectious diseases (e.g., reduced fecundity and increased mortality rates) constitute important selection pressures on individuals and have driven the evolution of sophisticated physiological defenses through complex immune systems (*2*). In addition, many species also exhibit behavioral strategies (e.g., grooming avoidance of parasitized conspecifics and selective foraging to avoid contaminated grazing areas) that can serve as the first line of defense against directly or environmentally transmitted pathogens (*3*–*6*).

Sexually transmitted infections (STIs) represent a particularly interesting case, as they are tightly linked to mating behavior and consequently to reproductive fitness. Costs associated with STIs include chronic infections with low recovery rates, reduced offspring survival, sterility, and costly immune defenses (*7*, *8*). However, unexpectedly, little is known whether individuals avoid mating with infectious partners.

**Fig. 1:**
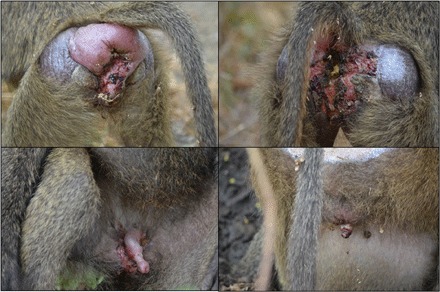
Genital ulcerations caused by *Treponema pallidum*. Clinical signs of infection in adult females (top) and males (bottom) of our study group. Photo credit: F. M. D. Paciência, German Primate Center.

Syphilis, a disease caused by the bacterium *Treponema pallidum* subsp. *pallidum*, is one of the most common STIs in humans. Individuals become infected by direct contact, usually sexual, with an infectious lesion (i.e., primary chancre) in the genital area (*9*). In Tanzania, at Lake Manyara National Park (LMNP), olive baboons (*Papio anubis*) are infected with a closely related bacterium [*T. pallidum* subsp. *pertenue* (*TPE*) (*10*, *11*)]. Other nonhuman primates (NHPs) such as yellow baboons (*Papio cynocephalus*), blue monkeys (*Cercopithecus mitis*), and vervet monkeys (*Chlorocebus pygerythrus*) are also found infected (*11*). In humans, *TPE* is known to cause yaws, a nonvenereal disease that spreads via skin-to-skin contact and causes skin ulcers in different body regions [e.g., on the face, arms, and legs (*9*)]. In contrast, clinical signs of *TPE* in NHPs in Tanzania mainly manifest themselves in the anogenital region, resembling a syphilis-like infection. Infected individuals can be identified by the appearance of genital ulcerations that lead to partial or complete mutilation of the external genitalia (Fig. 1) (*10*, *12*, *13*). Nevertheless, in rare cases, facial lesions can also be observed among TPE-infected baboons (*13*). *T. pallidum* is very sensitive to temperature changes and desiccation outside of its host (*9*). Therefore, genital skin-to-skin transmission is a highly effective transmission pathway as the genital area provides a constant moist environment compared to other regions of the body. This, together with the high frequency of observed genital ulcerations in sexually mature NHPs in Tanzania, suggests that *TPE* in those is most likely sexually transmitted (*10*, *12*).

Every time a susceptible individual mates with an infectious partner, it exposes itself to pathogens and subsequent loss of fitness, but the overall benefit of avoidance behavior might be counterbalanced by the cost of missed mating opportunities. Theoretical models have analyzed the potential impacts that STIs might have on mate choice and on the evolution of mating systems (*14*–*17*), but empirical data on animal host–STI interactions are lacking.

In this study, we investigated the sexual behavior of a wild olive baboon population to test whether the genital health status (GHS) had an impact on mate choice and mating behavior. We hypothesized that olive baboons at LMNP can discriminate between genitally ulcerated and non-ulcerated individuals and consequently adapt their mating behavior according to the GHS of the sexual partner. We assumed that individuals were infectious when genital ulcers were present, as is the case in humans infected with *T. pallidum* (*9*). We postulated that (i) non-ulcerated individuals avoid mating with ulcerated conspecifics and that (ii) mating patterns (i.e., the number of mating attempts and copulations) of ulcerated individuals are reduced when compared to non-ulcerated individuals.

## RESULTS

### Frequency of genital ulcers

The prevalence of genitally ulcerated individuals, determined visually, remained almost stable throughout the 18-month study period. At the end of the study, 44% of the 53 adult and subadult females and 47% of the 35 adult and subadult males displayed genital ulcers and were therefore considered as infectious (Fig. 1). Six baboons (three males and three females) that showed no ulcers at the beginning of the study developed genital ulcers, and no recovery was observed. No clinical signs were detected in infants, but five juveniles (one female and four males) were observed with genital ulcerations (of an estimated 70 immature individuals). Genital ulcers were observed in 40% of the females participating in sexual behaviors (of *n* = 32 cycling and well-habituated females) and in 53% of the males (*n* = 35).

### Mating patterns

**Fig. 2:**
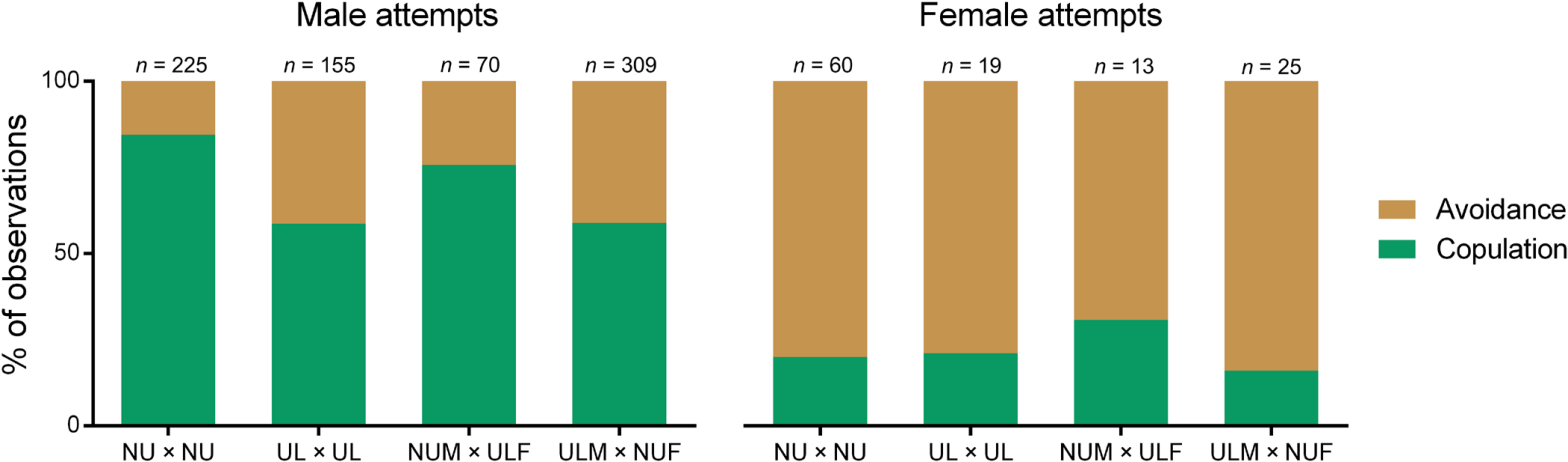
Percentage of male and female mating attempts according to their GHS. Each attempt could result in either copulation (green bar) or avoidance (brown bar). The percentage of observations is shown on the *y* axis. The attempts between individuals according to their GHS are shown on the *x* axis. NU × NU (non-ulcerated × non-ulcerated), UL × UL (ulcerated × ulcerated), NUM × ULF (non-ulcerated male × ulcerated female), ULM × NUF (ulcerated male × non-ulcerated female). Sample sizes are shown at the top of each bar.

**Fig. 3:**
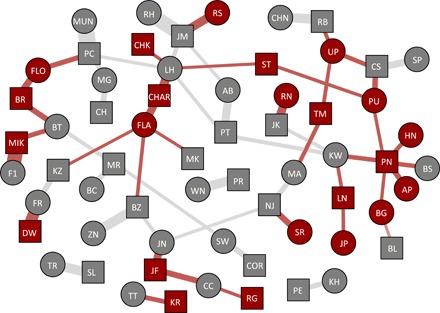
Olive baboon mating network. Nodes represent individual baboons and are colored according to each baboon’s GHS. Red nodes represent ulcerated individuals and gray non-ulcerated. Individuals who switched GHS are colored according to the status which had a higher number of copulations. Squares and circles represent males and females, respectively. Edges are weighted by the number of copulations among dyads and respective focal time. Edge colors correspond to the pairwise GHS of the nodes they connect, with red edges representing copulations where at least one of the individuals is ulcerated, while gray edges represent copulations between non-ulcerated individuals.

The majority of the mating behaviors (91% of *n* = 876 mating attempts) occurred when a female was in peak estrus, i.e., when a female’s sexual swelling was at its maximum size. Males initiated most of the mating attempts (86%; Fig. 2). We observed 876 mating attempts between 32 females and 35 males (Fig. 3) of which 540 resulted in copulations. Males with genital ulcers performed fewer pelvic thrusts during copulations than non-ulcerated males (median of 5 and 8 pelvic thrusts, respectively; Mann-Whitney *U* test, *W* = 63.948; *P* < 0.001).

Among the 32 focal females (table S1), we observed an average of 2.94 cycles (range, 1 to 6) within the 18-month study period. During this period, each female had an average of 1.8 mating partners (range, 1 to 6; Fig. 3), with a median of 1 partner per 3 cycles. Females spent 95% of the maximum sexual swelling time in consortship with a male partner.

Contrary to our expectations, the number of mating attempts observed for each possible female-male dyad revealed no significant effect of the GHS of the male or the female (fig. S1), indicating a lack of support for the hypothesis that GHS status affects mating attempts. This result was obtained irrespective of whether the mating attempts were initiated by the males [model TA-1 (total attempt–1); Table 1] or by the females [model TA-2 (total attempt–1); Table 1]. However, as we predicted, acceptance of mating attempts was influenced by the GHS of both baboons in a dyad. Attempts initiated by males [model SA-1 (successful attempt–1); Table 2] were significantly less likely to result in copulations if either the male or the female was genitally ulcerated (male GHS, *P* = 0.007; female GHS, *P* = 0.021). The odds of a successful male-initiated copulation were 3.2 times higher if the male was non-ulcerated versus ulcerated and 3.1 times higher if the female was non-ulcerated versus ulcerated. In contrast, acceptance of mating attempts initiated by females [model SA-2 (successful attempt–2); Table 2] was not significantly influenced by either male or female GHS (male GHS, *P* = 0.55; female GHS, *P* = 0.37), despite the high number of mating avoidance events by males (Fig. 2).

**Table 1:** Total attempt models. Weibull mixed-effects models evaluating whether the total number of mating attempts is influenced by the dyad’s GHS of the male and/or the female. Mating attempts initiated by males and females are shown in TA-1 and TA-2, respectively. Given are posterior means, SDs, 2.5 and 97.5% confidence intervals (CIs), and effective sample sizes (*n*). Rhat = 1 (model convergence) for all parameters. Models were run using four chains, each with 2000 iterations, a burn-in of 1000, and thinning set to 1.

**TA-1 model**					
**Term**	**Mean**	**SD**	**CI lower**	**CI upper**	***n* (effective)**
Intercept *q*	−3.40	0.24	−3.89	−2.94	2799
Intercept β	−2.11	0.20	−2.53	−1.75	2550
Main effects:					
GHS females *q*	0.04	0.28	−0.52	0.59	4000
GHS females β	0.17	0.24	−0.31	0.64	4000
GHS males *q*	0.37	0.28	−0.18	0.92	2942
GHS males β	0.07	0.24	−0.40	0.56	3025
Random effects:					
Male ID *q*	0.24	0.17	0.01	0.63	1803
Male ID β	0.14	0.11	0.01	0.40	2027
Female ID *q*	0.17	0.13	0.01	0.47	2424
Female ID β	0.11	0.09	0.00	0.33	4000
**TA-2 model**					
**Term**	**Mean**	**SD**	**CI lower**	**CI upper**	***n* (effective)**
Intercept *q*	−3.56	0.28	−4.15	−3.05	4000
Intercept β	−1.32	0.25	−1.80	−0.83	4000
Main effects:					
GHS females *q*	0.12	0.32	−0.51	0.73	4000
GHS females β	0.50	0.34	−0.18	1.17	4000
GHS males *q*	−0.21	0.35	−0.92	0.44	4000
GHS males β	−0.27	0.35	−1.00	0.37	4000
Random effects:					
Male ID *q*	0.43	0.27	0.02	1.02	1222
Male ID β	0.19	0.16	0.01	0.59	1982
Female ID *q*	0.24	0.18	0.01	0.65	2192
Female ID β	0.32	0.22	0.01	0.83	1385

**Table 2:** Successful attempt models. GLMMs evaluating whether the success of a mating attempt (i.e., likelihood of copulation) is influenced by the dyad’s GHS of the male and/or the female. Mating attempts initiated by males and females are shown in SA-1 and SA-2, respectively. Estimates, SEs, df, and 2.5 and 97.5% confidence intervals are shown for fixed effects. Significant variables (*P* < 0.05) are shown in bold. Intercept with reference category for non-ulcerated individuals.

**SA-1 model**							
**Term**	**Estimate**	**SE**	**CI lower**	**CI upper**	***X*^2^**	**df**	***P***
Intercept	2.371	0.477	1.628	3.326	*	*	*
GHS females	−1.127	0.480	−1.962	−0.325	7.187	1	**0.007**
GHS males	−1.167	0.481	−2.151	−0.228	5.315	1	**0.021**
**SA-2 model**							
Intercept	−1.542	0.469	−2.782	−0.735	*	*	*
GHS females	0.577	0.647	−0.738	1.983	0.794	1	0.373
GHS males	−0.396	0.657	−1.727	1.055	0.348	1	0.555

*Not shown due to very limited interpretation

## DISCUSSION

In animal populations, mating is tightly linked to fitness maximization, and therefore, the selection of healthy partners is crucial to avoid fitness costs associated with STIs. We examined whether wild olive baboons avoided mating with individuals affected by conspicuous genital ulcers caused by *T. pallidum*. Our results demonstrate a significant effect of clinically apparent *T. pallidum* infection on the mating behavior of female baboons. Females were more likely to avoid copulation if approached by males with ulcerated genitals, indicating behavioral avoidance of diseased conspecifics. NHP females are often more susceptible to STI acquisition than males, as is observed in humans (*2*). Female-biased disease risk avoidance is widespread across species, and infections can significantly affect mate choice (*18*). In general, males that are infected with directly transmitted parasites (e.g., nematodes, protozoa, bacteria, and viruses) are less preferred mating partners by their conspecific females (*18*). As compared to men, women also exhibit a higher level of disgust toward potential disease threats (e.g., contaminated environment and spoiled food) and show a higher tendency to avoid infected sexual partners [e.g., individuals exhibiting genital lesions (*19*, *20*)]. The results of our study are in accordance with this generalized higher disgust reported in females since mating with STI-infected partners can entail greater costs to females due to obligatory investment in gestation, lactation, and infant rearing.

We also found that avoidance by females was more frequent if females themselves had ulcerated genitals. The progressive scarification of the genitalia due to infection can lead to a permanently open state of the vagina and anus, which increases urogenital infections (i.e., promoting miscarriages) and risk of dystocia. Thus, in addition to affecting the mate choice of non-ulcerated individuals, *T. pallidum* appears to affect mating behavior of ulcerated females, as genital ulcers are likely painful and potentially cause afflicted females to refrain from mating.

While we found no evidence to support the hypothesis that males avoid mating opportunities based on their own or their partner’s GHS, genitally ulcerated individuals performed significantly fewer pelvic thrusts during copulation than non-ulcerated males. Fewer pelvic thrusts might result in no or fewer ejaculation events. In addition, some symptomatic individuals were observed showing unusual body contractions with simultaneous vocalizations (e.g., kecking) during urination. As observed in females, genital ulcers seem to be equally painful for males, thus, to a certain extent, ulcerated males might also incur fitness costs.

STIs have been hypothesized to constitute a key selection pressure in shaping the evolution of mating strategies (*14*, *15*, *21*). A model based on a human hunter-gatherer population indicated that a high prevalence of STIs in large group sizes could foster the emergence of socially imposed monogamy (*22*). Cycling females of yellow and olive baboons have been reported to copulate with most of the males comprising their group (*23*, *24*). In contrast, in our study, female olive baboons only consorted and mated with few partners (average of 1.8), despite the large pool of males available (*n* = 35). While the reason for this low promiscuity is unclear, and studies reporting on olive baboon promiscuity are scarce, it would be important to investigate whether factors such as group size, pathogen incidence, pathogen virulence, and fitness consequences due to *T. pallidum* exert a selective pressure on our baboon population, which, in return, could lead to altered mating strategies.

A potential limitation of our study is that we were unable to collect data on male dominance rank; however, we think it is unlikely that including rank data in our models would significantly affect our findings. First, the vast majority of sexually mature males (83%) in our study group engaged in copulations and established consortships with peak-estrous females, indicating a lack of mating skew according to rank. Second, despite the fact that dominance rank has been considered a predictor of mating success in yellow and olive baboons, it is also a subject of high variation among groups and individuals, and it appears to depend on multiple factors (*25*, *26*). This highly contrasts with chacma baboons, where rank and access to estrous females are strongly connected (*27*). In addition, in yellow and olive baboons, group size and composition are important determinants of mating access among male baboons: High-ranking males tend to lose their monopoly over cycling females in large groups (*28*), and male dominance ranks are less pronounced in large groups with many cycling females (*29*). Given that olive baboon groups at LMNP are extraordinarily large (averaging approximately 150 individuals), we consider that rank does not play a major role regarding access to reproductive females in our study population. Instead, our results indicate that partner preference is crucially important at LMNP, as we observed cycling females maintaining a consistent male partner across consecutive estrus cycles.

Empirical studies on mating behavior in relation to STIs are scarce and mainly confined to arthropods. In these, no evidence of mating avoidance or discrimination between healthy and sick conspecifics has been found (*30*–*32*). Fitness disadvantages, disgust, and pain anticipation might be influencing the mating patterns of our group. Our study reporting on the ability of a long-lived vertebrate to discriminate among mating partners according to health status sheds light on how sexually transmitted pathogens can markedly shape mating dynamics in a group of NHPs. Last, given that *T. pallidum* is present in humans in overlapping areas with infected NHPs (*11*), the investigation of the underlying mechanisms affecting pathogen transmission is of the uttermost importance to mitigate health risks for both human and wildlife communities.

## MATERIALS AND METHODS

### Study site and subjects

Fieldwork was conducted at LMNP, Northern Tanzania (3°28′ S 35°46′E), during two field seasons (April to December) in 2015 and 2016. LMNP is a small protected area (approximately 580 km^2^) with almost 220 km^2^ of lake coverage. In 2016, we conducted a survey estimating the population size of the LMNP baboons in the park excluding the recently added area of the Marang forest (250 km^2^). The population was estimated to consist of approximately 5200 olive baboons, and individuals with ulcerated genitals were observed in most groups within LMNP.

We habituated our study group (*n* ≈ 170 baboons) over a 4-month period before data collection. To enhance location of the group, we radio-collared three adult females (Advanced Telemetry Systems Inc., Isanti, MN, USA). Proceedings on immobilization and anesthesia are described in (*13*). Our analyses focused on 26 adult and 11 subadult females (after excluding data of females observed <1.5 hours) and 28 adult and 7 subadult males. Age categories were defined as in (*33*). Adult males were identified by their large body size and fully developed secondary sexual traits; subadult males were larger than females but lacked secondary sexual characteristics (e.g., large shoulder mane and elongated canines). Adult females were identified as individuals that have reached full body size, whereas subadult females were smaller and lacked elongated nipples (but were already cycling).

### Genital health status

Within our study group, baboons were categorized as either “genitally ulcerated” or “non-ulcerated,” based on their GHS, using macroscopic visual cues [as in (*10*)]. In both males and females, genital ulcerations were observed ranging from small-medium ulcers to severe necrotizing dermatitis and mutilation of the outer genital structures (Fig. 1).

### Behavioral data

We conducted focal follows (*34*) from dawn to dusk on subadult and adult females. We aimed for full-day focal follows, but if a focal individual was out of sight for more than 10 min, then another baboon was selected. We recorded 597 hours of observation data, with an average of 16.40 ± 10.02 hours (mean ± SD; range, 1.50 to 39.00 hours) per focal female. We prioritized following females in their peak estrus [denoted by maximal tumescence of the anogenital area and bright pink color (*35*)] to maximize the number of mating events observed. We collected data on the sexual behavior of focal females and their partners (table S1), recording when dyads participated in consortships in which a male maintained close proximity to a female and attempted to prevent other males from mating with her (*24*). In addition, we collected data on the frequency and success of mating attempts led by either males or females. Male-led mating attempts were identified as a male trying to mount a female with the performance of pelvic thrusts (*36*). Attempts led by females were documented when a female presented her perineum to a male while lifting her tail (*37*). Attempts by either sex were considered successful if they resulted in copulation. Because of the loss of the corpus penis or phimosis, some males were unable to engage in intromission and/or ejaculation, precluding our ability to use these behaviors to define successful mating attempts. An unsuccessful attempt was defined as a female rejecting a male’s attempt to mount (e.g., by sitting or fleeing) or a male refraining from mounting a female after she presented to him. Behavioral data were recorded on a hand-held device (Samsung Galaxy Hand Note) in the field using Pendragon 5.1.2 software (Pendragon Software Corporation, USA) and transferred daily onto computers for error checking and data storage.

### Statistical analyses

#### *Total attempt model*

We first investigated whether the number of mating attempts was affected by the GHS of a dyad. To examine this, we developed a dataset that indicated the corresponding number of attempts made by the male and female of every possible dyad in the study group, along with their respective GHSs. Including every possible dyad allowed us to consider all potential mating attempts, as mating could theoretically occur between any cycling female and any male in the group. This dataset included a high proportion (96.3%) of zero attempt data, with data for nonzero attempts showing extremely high variance (σ^2^ = 149.9). Distributions commonly used in instances of zero inflation [e.g., zero-inflated Poisson or negative binomial distributions (*38*)] were a poor fit for our data; however, the discrete Weibull distribution (*39*) proved to be a good fit (fig. S2). In particular, the discrete Weibull distribution is highly flexible in modeling under- and overdispersed data relative to the Poisson distribution (*40*). The discrete Weibull probability mass function is defined as

$$P(X=x)=q^{x^{\beta }}-q^{{\left(x+1\right)}^{\beta }}$$

with positive shape parameter β and parameter *q* satisfying 0 < *q* < 1. As no current R packages offered the functionality to fit mixed-effects models with a discrete Weibull distribution, we used the Bayesian programming language “Stan” to develop discrete Weibull mixed-effects models (*41*). We built two models: one predicting the number of attempts led by males (model TA-1; Table 1) and a second for attempts led by females (model TA-2; Table 1). Each model assumed that *q* and β were a linear combination of the male GHS and the female GHS (fig. S2). Both linear predictors also included male and female identities as crossed random effect variables affecting model intercepts. We used uninformative or weakly informative priors. Fixed effect coefficients had normally distributed priors and SDs equal to 100. Random effects were assumed to be normally distributed with SDs sampled from Cauchy prior distributions with location parameters equal to zero and scale parameters equal to 25. All models were fitted using R v 3.4.4 (*42*) with the “Rstan” package (*43*).

#### *Successful attempt model*

Here, we examined whether the success of a mating attempt is affected by the GHS of the dyad. Our dataset included the success (1/0) of each mating attempt observed, along with the GHS of the male and female involved in the mating attempt. We built two generalized linear mixed models (GLMMs) (*44*): one model for attempts led by males (model SA-1; Table 2) and a second model for attempts led by females (model SA-2; Table 2). Each model assumed that the probability of a successful mating event depended on the GHS of the male and GHS of the female. Both models had a binomial error structure with a logit link function and included the individual and pair identities as random effects. In addition, we included the focal observation time as an offset term to account for variation in observation effort among cycling females. The interactions between male and female GHS were nonsignificant and were excluded from the final models. Models were run in R v 3.4.4 (*42*) with the lme4 package v 1.1-15 (*45*), and *P* values are shown based on likelihood ratio tests of individual fixed effects [function drop1 with argument *test* set to “Chisq”, (*46*)].

#### *Network visualization*

We constructed a mating network (Fig. 3) using a force-directed (Fruchterman-Reingold) layout in R v 3.4.4 (*42*) with the igraph package v 1.2.2. Connections between nodes (edges) were weighted according to each dyad’s copulation rate (i.e., the number of copulations after controlling for the female’s observation time). To aid in visualizing connections among nodes, we made minor adjustments to the final network graphic (e.g., preventing overlapping nodes).

## Supplementary Material

http://advances.sciencemag.org/cgi/content/full/5/12/eaaw9724/DC1

Download PDF

Mating avoidance in female olive baboons (Papio anubis) infected by Treponema pallidum

## SUPPLEMENTARY MATERIALS

Supplementary material for this article is available at http://advances.sciencemag.org/cgi/content/full/5/12/eaaw9724/DC1 Fig. S1. Posterior probabilities with *q* and β estimation parameters. Fig. S2. Observed and predicted values using a discrete Weibull distribution. Table S1. Focal females and their respective mating partners.
