# Supplementary material for: Mating avoidance in female olive baboons (Papio anubis) infected by Treponema pallidum
Source: Sci Adv. 2019 Dec 4;5(12):eaaw9724. doi: 10.1126/sciadv.aaw9724 (PMC6892622; doi:10.1126/sciadv.aaw9724)
Supplement: http://advances.sciencemag.org/cgi/content/full/5/12/eaaw9724/DC1 [file supp_5_12_eaaw9724__index.html]

Science Advances | Science AdvancesAAASSearchScience AdvancesMenu

## Supplementary Materials

**This PDF file includes:**

- Fig. S1. Posterior probabilities with *q* and β estimation parameters.
- Fig. S2. Observed and predicted values using a discrete Weibull distribution.
- Table S1. Focal females and their respective mating partners.

Download PDF

**Files in this Data Supplement:**

- Adobe PDF - aaw9724\_SM.pdf
