# Supplementary material for: Mating avoidance in female olive baboons (Papio anubis) infected by Treponema pallidum
Source: Sci Adv. 2019 Dec 4;5(12):eaaw9724. doi: 10.1126/sciadv.aaw9724 (PMC6892622; doi:10.1126/sciadv.aaw9724)
Supplement: Download PDF [file aaw9724_SM.pdf]

Science Advances
AAAS

advances.sciencemag.org/cgi/content/full/5/12/eaaw9724/DC1

Supplementary Materials for

Mating avoidance in female olive baboons (*Papio anubis*) infected by *Treponema pallidum*

F. M. D. Paciência*, J. Rushmore, I. S. Chuma, I. F. Lipende, D. Caillaud, S. Knauf, D. Zinner

*Corresponding author. Email: fpaciencia@dpz.eu

Published 4 December 2019, *Sci. Adv.* **5**, eaaw9724 (2019)
DOI: 10.1126/sciadv.aaw9724

This PDF file includes:

Fig. S1. Posterior probabilities with q and β estimation parameters.
Fig. S2. Observed and predicted values using a discrete Weibull distribution.
Table S1. Focal females and their respective mating partners.

Supplementary Materials

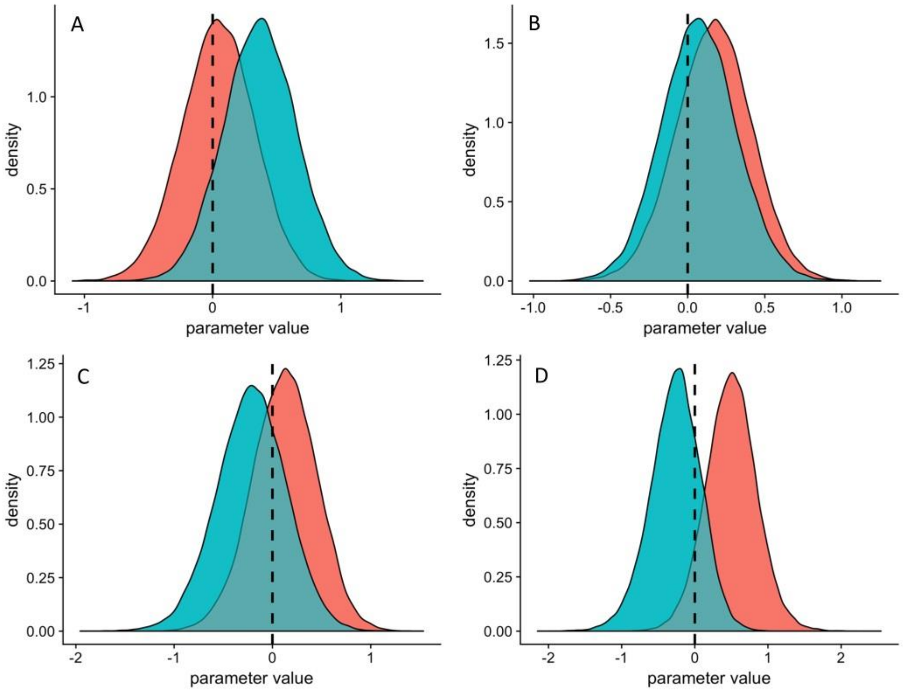

Fig. S1. Posterior probabilities with *q* and β estimation parameters. Posterior probability plots estimating the discrete Weibull distribution shape parameters, q (**A**, **C**) and β (**B**, **D**), in relation to the male attempts model (**A**, **B**) and female attempts model (**C, D**). Male genital health status (GHS) and female GHS fixed effects are displayed as blue and red, respectively. The posterior probabilities for both male and female GHS fixed effects cross the zero threshold in each panel, showing no support for the hypothesis that GHS affects the number of mating attempts.

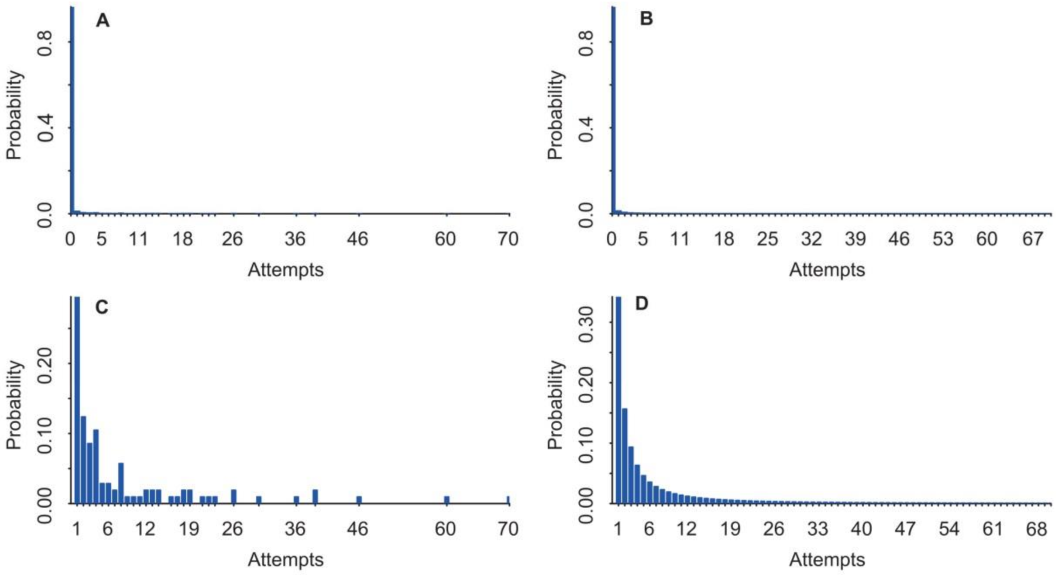

Fig. S2. Observed and predicted values using a discrete Weibull distribution. Observed data (left) as compared to the model predicted values (right) with only q and β (shape and scale) parameters. **A**, **B** shows the full dataset and demonstrates the model's ability to predict the zero-attempt data. **C**, **D** excludes zero-attempt data (values > 1) and demonstrates the model's ability to predict the non-zero attempt data.

Table S1. Focal females and their respective mating partners.

female	age class	female GHS	male mating partner	age class	male GHS	focal time (h)
AB	subadult	non-ulcerated	JM	subadult	non-ulcerated	19.39
AB	subadult	non-ulcerated	PT	adult	non-ulcerated	19.39
AP	adult	ulcerated	PN	adult	ulcerated	4.96
BC	subadult	non-ulcerated	MR	adult	non-ulcerated	10.5
BG	adult	ulcerated	BL	adult	non-ulcerated	24.64
BG	adult	ulcerated	PN	adult	ulcerated	24.64
BS	adult	non-ulcerated	PN	adult	ulcerated	15.32
BS	adult	non-ulcerated	RD	adult	ulcerated	15.32
BT	adult	non-ulcerated	BR	adult	ulcerated	5.79
BT	adult	non-ulcerated	MIK	adult	ulcerated	5.79
BT	adult	non-ulcerated	MR	adult	non-ulcerated	5.79
CC	adult	non-ulcerated	JF	subadult	ulcerated	16.70
CC	adult	non-ulcerated	RG	subadult	ulcerated	16.7
DH	adult	non-ulcerated	FE	adult	non-ulcerated	1.64
CHN	adult	non-ulcerated	RB	adult	non-ulcerated	8.05
F1	adult	non-ulcerated	MIK	adult	ulcerated	5.86
FLA	adult	ulcerated	BZ*	adult	ulcerated	20.28
FLA	adult	ulcerated	CHAR	adult	ulcerated	20.28
FLA	adult	ulcerated	KZ	adult	non-ulcerated	20.28
FLA	adult	ulcerated	MK	adult	non-ulcerated	20.28
FLO	adult	ulcerated	BR	adult	ulcerated	14.42
FLO	adult	ulcerated	PC*	adult	ulcerated	14.42
FR*	adult	non-ulcerated	DW	adult	ulcerated	11.02
FR*	adult	ulcerated	KZ	adult	non-ulcerated	18.37
HN	adult	ulcerated	PN	adult	ulcerated	27.02
IN	adult	ulcerated	†	†	†	11.31
JN	subadult	non-ulcerated	BZ*	adult	ulcerated	28.95
JN	subadult	non-ulcerated	JF	subadult	ulcerated	28.95
JN	subadult	non-ulcerated	NJ	adult	non-ulcerated	28.95
JD	adult	ulcerated	RB	adult	non-ulcerated	10.33
JP*	adult	non-ulcerated	†	†	†	5.50
JP*	adult	ulcerated	LN	adult	ulcerated	23.06
KH	subadult	non-ulcerated	PE	subadult	non-ulcerated	9.72
KN	subadult	non-ulcerated	†	†	†	2.43
KW	adult	non-ulcerated	JK	subadult	non-ulcerated	22.35
KW	adult	non-ulcerated	LN	adult	ulcerated	22.35
KW	adult	non-ulcerated	PN	adult	ulcerated	22.35
KW	adult	non-ulcerated	PT	adult	non-ulcerated	22.35
LH*	subadult	non-ulcerated	CHAR	adult	ulcerated	13.03
LH*	subadult	non-ulcerated	CHK	adult	ulcerated	13.03
LH*	subadult	non-ulcerated	JM	subadult	non-ulcerated	13.03
LH*	subadult	non-ulcerated	PC*	adult	ulcerated	13.03
LH*	subadult	non-ulcerated	PT	adult	non-ulcerated	13.03

LH*	subadult	ulcerated	ST	adult	ulcerated	25.98
LH*	subadult	ulcerated	CHAR	adult	ulcerated	25.98
LH*	subadult	ulcerated	JM	subadult	non-ulcerated	25.98
MA	adult	non-ulcerated	NJ	adult	non-ulcerated	25.8
MA	adult	non-ulcerated	TM	adult	ulcerated	25.8
MG	adult	non-ulcerated	CH	adult	non-ulcerated	18.86
MG	adult	non-ulcerated	PC*	adult	ulcerated	18.86
MUN	subadult	non-ulcerated	PC*	adult	non-ulcerated	1.50
PU	adult	ulcerated	CS*	subadult	ulcerated	32.63
PU	adult	ulcerated	PN	adult	ulcerated	32.63
PU	adult	ulcerated	ST	adult	ulcerated	32.63
RH	subadult	non-ulcerated	JM	subadult	non-ulcerated	10.23
RN	subadult	ulcerated	JK	subadult	non-ulcerated	4.97
RS	subadult	ulcerated	JM	subadult	non-ulcerated	13.66
SP	adult	non-ulcerated	CS*	subadult	ulcerated	26.78
SR	adult	ulcerated	NJ	adult	non-ulcerated	5.20
SW	subadult	non-ulcerated	COR	subadult	non-ulcerated	26.77
SW	subadult	non-ulcerated	MR	adult	non-ulcerated	26.77
TR	adult	non-ulcerated	SL	adult	non-ulcerated	7.11
TT	adult	non-ulcerated	KR	adult	ulcerated	5.81
UP	adult	ulcerated	CS*	subadult	ulcerated	25.45
UP	adult	ulcerated	RB	adult	non-ulcerated	25.45
UP	adult	ulcerated	TM	adult	ulcerated	25.45
WN	adult	non-ulcerated	PR	adult	non-ulcerated	21.57
ZN	adult	non-ulcerated	BZ*	adult	ulcerated	14.11

Focal time is given in decimal hours.
*Individuals who switched from category “non-ulcerated” in Season 1 to category “ulcerated” in Season 2. Females are listed with two observation times respectively, one for each season.
† No mating observed.
